# Urine Scent Marking (USM): A Novel Test for Depressive-Like Behavior and a Predictor of Stress Resiliency in Mice

**DOI:** 10.1371/journal.pone.0069822

**Published:** 2013-07-16

**Authors:** Michael L. Lehmann, Claire E. Geddes, Jennifer L. Lee, Miles Herkenham

**Affiliations:** Section on Functional Neuroanatomy, National Institute of Mental Health, National Institutes of Health, Bethesda, Maryland, United States of America; Oregon Health and Science University, United States of America

## Abstract

Decreased interest in pleasurable stimuli including social withdrawal and reduced libido are some of the key symptomatic criteria for major depression, and thus assays that measure social and sexual behavior in rodents may be highly appropriate for modeling depressive states. Here we present a novel approach for validating rodent models of depression by assessing male urine scent marking (USM) made in consequence to a spot of urine from a proestrous female. USM is an ethologically important form of sexual communication expressed by males to attract females. The expression of this behavior is highly sensitive and adaptive to environmental cues and social status. We hypothesized that male USM behavior offers a naturalistic measure of social motivation that can be used to evaluate hedonic behaviors relevant to the study of mood disorders. We demonstrated that 1) adult male mice displayed a strong preference for marking proestrous female urine with a high degree of specificity, 2) exposure to chronic social defeat profoundly decreased USM whereas exposure to environmental enrichment increased USM, 3) the standard antidepressant fluoxetine reversed declines in USM induced by social defeat, 4) USM behavior closely correlated with other hedonic measures, and 5) USM scores in non-stressed mice predicted behavioral outcomes after defeat exposure such that mice displaying high preference for marking female urine prior to social defeat showed behavioral resiliency after social defeat. The findings indicate that the USM test is a sensitive, validated measure of psychosocial stress effects that has high predictive value for examination of stress resiliency and vulnerability and their neurobiological substrates.

## Introduction

Progress in understanding the etiology of depression relies on studies using well-validated animal models that address underlying psychopathology and potential treatments. Social stress in humans is a main etiological factor for numerous psychopathologies including depression and anxiety [1], [2]. Hence, animal models that utilize psychogenic stress paradigms to induce depressive-like behavior have high translational relevance [3]–[5]. In the psychosocial defeat paradigm, rodents that accumulate social defeats show profound and enduring behavioral and endocrine changes similar to those seen in human mood disorders including reduced social interaction, anhedonia, and alterations in hypothalamic-pituitary (HPA) axis activity and endocrine metabolism [6]–[15]. Many of these changes are reversed by chronic but not acute antidepressant administration [16], [17]. Thus, the social defeat (SD) model exhibits face, construct, and predictive validity [18].

Assessment of the efficacy of stress paradigms such as SD requires tests of depressive-like behavior in validated behavioral assays. Currently used tests–the learned helplessness test (LH), forced swim test (FST), and tail suspension test (TST)–compare escape (active) versus immobility (passive) behaviors as a means for examining depressive-like behavior. These assays were initially developed to screen drugs for antidepressant activity [19], [20]. Whereas they are predictive of antidepressant activity after acute administration of the drugs, in human depression, it is well documented that the same drugs achieve efficacy only after weeks of daily administration. The immobility measures were not initially designed nor claimed to be reflective of depressive states that could be reversed by the drugs [21].

Diminished hedonic behavior is a defining feature of many psychiatric disorders, notably major depressive disorder (MDD), and so measures of hedonic or reward-seeking behaviors have convincing face validity. The sucrose/saccharin-preference test is one of the more widely used tests for anhedonia. However, sucrose consumption correlates with body weight, which is often altered in stressed animals compared to controls [22]. Furthermore, numerous tests have shown no reduction in preference after stress exposure, demonstrating poor reliability of this test (see [23] for review) [22], [24], [25]. Other operant tasks examining hedonic behaviors, such as the conditioned place preference test, which depends heavily on learning and memory, and electrical or chemical intra-cranial self-stimulation, are complex, require extensive training periods and surgery, and are sensitive to altered performance. The confounding effects inherent in these tasks have necessitated the development other more natural measures of reward-related behavior, such as social interaction [26] and sexual behavior.

A good measure of depressive-like states should have ethological relevance, correlate with other accepted measures, and be simple, non-invasive, and accurately quantifiable. Here, we present a novel, accurate, and sensitive approach for validating rodent models of depression by assessing male urine scent marking (USM) relative to a spot of urine from a proestrous female. Social communication in rodents occurs primarily through olfactory cues. Scent marking in mice, through the deposition of urinary pheromones, supports territory demarcation [27], reproductive competition [28], [29], and sexual advertisement [30]–[32]. Contact with chemosensory cues in urine induces profound changes in physiology and behavior, including accelerating puberty, blocking pregnancy, and inhibiting aggressive behaviors [31]. Adult male mice actively scent mark in physical proximity to adult female urine to advertise their identity and dominance over other mice [30]–[32]. They are more attracted to urine from sexually receptive females in proestrus than those in diestrus [33]. More recent experiments have shown that female urine activates reward centers of the male rodent brain [34]. Thus the males’ interest in female urine can be interpreted as pleasurable. Scent marking is also a sexually motivated behavior expressed by males to attract females [29], [30], and reduced territorial marking may be considered maladaptive in that the male that does not claim a territory is less likely to procreate [30], [35].

Several scent marking tasks are already in use. One is performed in an open field, and the amount of territorial urine marking by male mice has been shown to be highly influenced by hierarchical status and by repeated social defeat [35]. However, these tests cannot be interpreted as demonstrating hedonic behavior because a rewarding cue is absent. Here we described a new task, the urine scent marking (USM) task, that utilizes female urine as a rewarding cue, and we hypothesized that the male’s scent marking activity in response to the female urine spot is a measure of the strength of hedonic behaviors. We found that social defeat (SD) stress significantly reduced marking behavior whereas enriched environmental (EE) housing increased scent marking in close proximity to the female urine spot. Furthermore, we observed that the ability of SD stress to reduce scent-marking behaviors is highly correlated with resilience to stress such that resilient mice showed pronounced scent marking after SD. Finally, we demonstrated that scent marking measured prior to SD is predictive of stress resiliency and thus can be used as a behavioral assay to examine neurobiological correlates of stress reactivity without the confounding effects of stress exposure.

## Materials and Methods

### Ethics Statement

All experiments were approved by the NIMH Institutional Animal Care and Use Committee and conducted in accordance with the NIH guidelines.

### Animals

Experiments were performed using CD-1 male and C57BL6/J (C57) male and female mice (aged 10–12 weeks) obtained from NIH/NCI/DCT (Frederick, MD). All mice were group-housed for one week after arrival. During experimental testing, female mice were group-housed, and male C57 mice were either singly or group-housed as determined by experimental design. All animals were housed in a reversed 12-h light/dark cycle (Lights OFF at 0900) and tested during the dark phase in red-light conditions. All treatment conditions were randomly assigned. Food and water were provided *ad libidum*.

### Female Urine Spotted in Open Field

**Figure 1:**
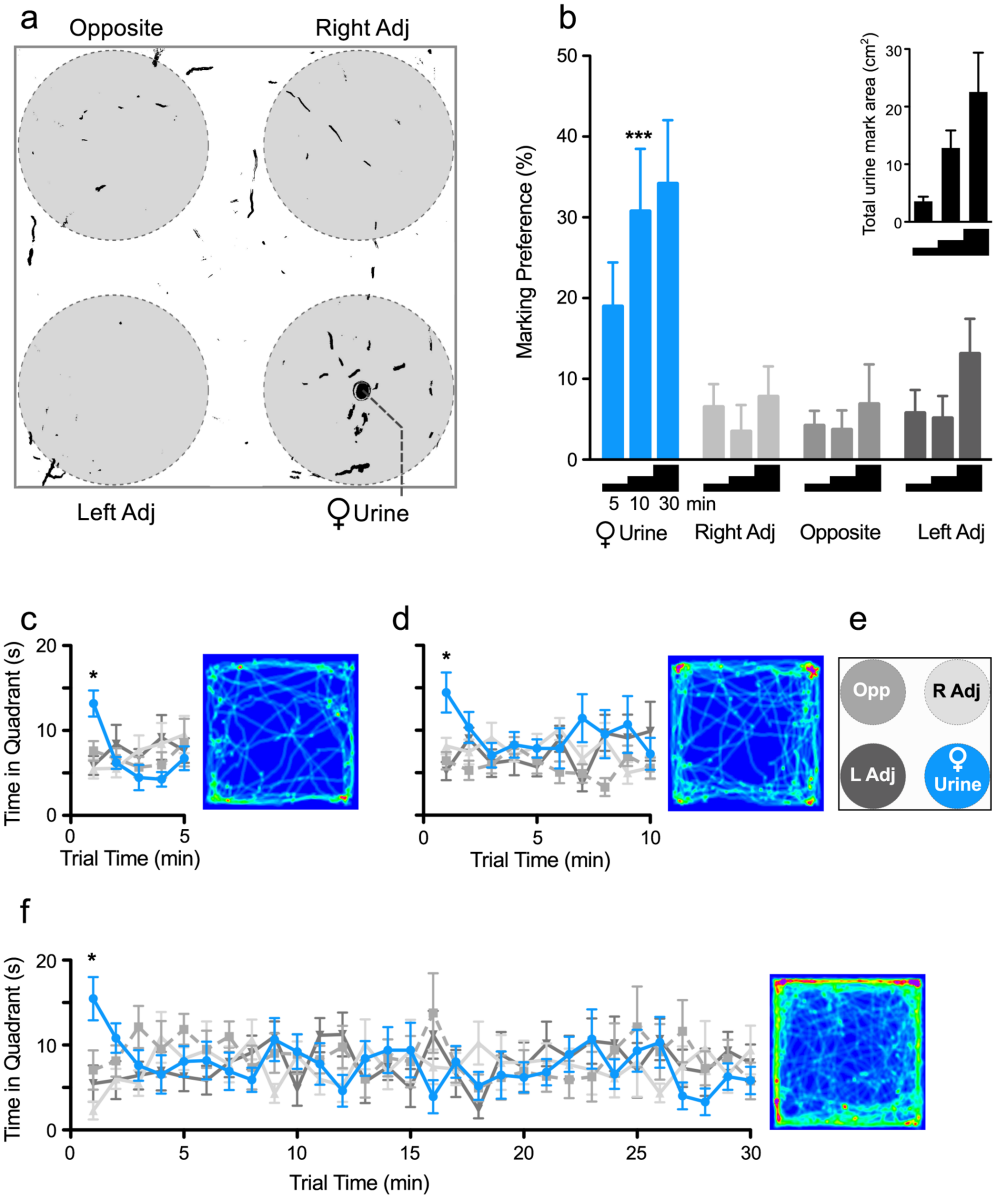
Male mice show a high preference for scent marking proestrous female urine. **a)** A binary image of urine scent marks deposited by a male mouse during a 10 min period is overlaid with a diagram showing analyzed areas and quadrant locations. Marking preferences are calculated by dividing the area of male urine marks within a specified region by the total area of marks deposited in the arena. **b)** Males were exposed to the urine scent marking task for 5, 10, or 30 min and showed a significant preference for marking the quadrant with female urine after 10-min trial. **Insert)** The area of urine scent marks in the total arena increased significantly across trial length. **c, d, f)** In a time course analysis of the duration spent in each of the four quadrants for the **c)** 5, **d)** 10, or **f)** 30 min trial periods, male mice show a preference for the quadrant containing female urine during the first min only. Densitometry plots that indicate location preferences during different trial lengths are shown to the right of each line graph. **e)** Legend for the line graphs and densitometry plots indicating quadrant location. Bars represent mean+SE (n = 10–12 per group), *p<0.05, ***p<0.001.

Female mice were maintained in a separate room, and the estrous stage was determined by visual inspection as previously described [36], [37] and confirmed by evaluating vaginal cytology [38], [39]. Experimental procedures used for collecting female urine and for blotting were adopted from previous reports [40]–[42]. Urine was collected from female mice in proestrus characterized by swollen pink vaginal opening and the presence of nucleated epithelial cells. Urine was obtained by applying gentle pressure to the bladder and collecting the urine with a blunt needle and syringe. Within five min of collection, 0.1 ml of female urine was blotted onto a 45.7 cm×45.7 cm acid-free paper sheet (Strathmore Sketch Paper 400 series, SLS Arts, New Orleans, LA) that was previously placed into an open field box (46 cm×46 cm). A 2-cm diameter spot of female urine was deposited in one corner of the arena, 10 cm from two adjacent edges of the paper (see Fig. 1a).

### Behavioral Conditions

All behavioral tests were performed in a testing room illuminated by red light at 14 lux. Experimental male mice were allowed to habituate in the testing room for one h prior to the behavioral task. After the female urine was absorbed by the paper and dried (∼ one min), male mice were placed in the center of the arena and allowed to freely explore and scent mark for 5, 10, or 30 min. Sessions were videotaped and later analyzed using automated behavioral tracking software (TopScan/ObjectScan, CleverSystems, Leesburg, VA,). The software reliably scores the location of the animal and the frequency and duration of sniffing through identification of the animals shape including nose, body and tail. The floor and walls were cleaned with 70% ethanol and dried after each session. Experiments using male urine as the stimulus cue were conducted in a similar fashion.

### Quantification of Urine Scent Marking (USM)

Urine scent marks were visualized using methods previously described [40]–[42]. Sheets were air-dried at least one h and then sprayed with ninhydrin (Tritech Forensics, Southport, NC), which stains amines in urine purple, and dried overnight. Dried sheets were photographed and analyzed using ImageJ software (http://rsb.info.nih.gov/ij/). Images were converted to binary. Circles 20-cm in diameter were digitally placed in each quadrant such that the edges of the large circle touched the two outer edges of the quadrant. This allowed one circle to be centered on the female urine spot. The area of male urine marking within each of the four circles was measured, as was the total area of marks within the arena. Preference of urine marking was calculated by dividing the area of urine marks in a 20-cm circle by the total area of urine marks within the arena and then multiplied by 100. Thus, dividing the area of marks within 10 cm of the center of the female urine spot (a circle 20 cm in diameter) by the total area of marks in the arena, then multiplying by 100 would provide the marking preference for the female urine quadrant. Investigation of the urine spot was defined as the duration spent sniffing the spot directly, a circle 2 cm in diameter centered over the urine spot. In Experiment 1, preference for marking was quantified for all four circles. In Experiments 2–4, only circles centered over the female urine spot were quantified for marking preference.

### Experimental Design

#### Experiment 1: Optimization of experimental conditions for USM

Due to the novelty of the USM task, optimization of trial length was assessed. Single-housed male mice were placed in the center of a scent-marked open field and allowed to explore for 5, 10, or 30 min. Following the trial, urine deposits were assayed as described above.

#### Experiment 2: Specificity for marking male and female urine

The second study examined scent-marking behavior in adult male mice exposed to urine spots from either a conspecific male or a proestrous female. Males were allowed to explore for 10 min, after which their scent marking was assayed.

#### Experiment 3: Effects of stress and environmental enrichment on USM

he third study examined the effects of housing environments and psychological stress exposure on urine-marking preference in male mice. To test the effects of housing environments, male mice were singly housed in either enriched housing environments (EE) or standard home-cage housing (HC). To test the effects of psychological stress, male mice were exposed to either daily restraint stress (RS) for two weeks or were exposed to SD stress for 5 min/day for two weeks. Urine marking preference was examined in all mice. Social interaction was examined one day after two weeks of SD or HC housing. A subset of SD- and HC-housed mice was further phenotyped for depressive-like behaviors. Defeated mice were not exposed to agonist interactions during the behavioral testing period.

#### Experiment 4: pharmacological rescue of behavioral pathology in socially defeated mice

A fourth study examined whether the defeat-induced reduction in scent marking behavior can be reversed by the selective serotonin reuptake inhibitor fluoxetine. Chronic, but not acute, treatment with antidepressants can remediate maladaptive behaviors in socially defeated mice [43], [44]. Chronic administration of antidepressants prior to [45], [46] or concurrent with [47] psychological stress exposure can reduce stress-induced changes in behavior. In this experiment, fluoxetine (20 mg/kg/day) (Sigma, Cat# F-132) or vehicle (0.9% NaCl) was administered i.p. (0.1 ml/10 gr body weight) once a day for three weeks to singly homecage-housed mice and was administered similarly throughout the remainder of the experiment. Mice were next randomly assigned to two weeks of either SD stress or continued homecage housing (2×2; Drug × Stress). At the cessation of the SD stress or continued homecage housing period, mice underwent a series of behavioral tests (one test/day) to assess the effects of SD and fluoxetine treatments. The USM test was carried out during the first day, followed by the SI task, saccharine preference, forced swim, and tail suspension. Defeated mice were not exposed to agonist interactions during the behavioral testing period.

#### Experiment 5: Urine marking as a predictor of stress resiliency

The last study tested urine marking as a predictor of stress resiliency in dominant vs. submissive mice. Co-habitating male mice readily form dominance hierarchies, and we hypothesized that social rank would influence USM behavior and would also predict resiliency to SD. To investigate social rank, we applied a tube test (described below) to six groups of male 8-week-old C57BL/6J mice that were co-housed four-per-cage for at least one week prior to start of testing. The tube test was applied every other day over a two-week period, and on each testing day, mice were tested pair-wise in a round-robin design. After social rank was assessed, mice were individually housed in standard home cage for two weeks to reduce acute effects of agonist interactions on subsequent behaviors. Mice were then exposed to the USM test and returned to their home cage. Twenty-four h later, each mouse was introduced into a cage housing a CD-1 male mouse (social defeat conflict cage, described below) and exposed to a single 5-min SD agonist interaction session. The defeated mouse remained in the partition cage, opposite the dominant animal for 24 h and then tested for stress resiliency in the social interaction task. Single defeat experiences result in long lasting neuroendocrine and behavioral consequences with rapid onset [11], [48]–[50] and where chosen as an initial screen for interactions between cage rank, marking behaviors, and stress resiliency.

### Housing Conditions

#### Standard homecage housing (HC)

Mice were singly housed for two weeks in polycarbonate cages (14.0 cm×35.5 cm×13.0 cm, Tecniplast, Montreal, Canada) that contained woodchip bedding and a cotton square nestlet in each. Bedding was replaced every seven days.

#### Enriched housing (EE)

Mice were singly housed for two weeks in polycarbonate cages (24.5 cm×40.5 cm×18.5 cm) that contained wood-chip bedding, nestlets, raised platforms, running wheel, and numerous paper and polycarbonate tubes of various sizes and shapes (Bio-Serv, Frenchtown, NJ). Polycarbonate tubes and wheels were washed with warm water, dried, and replaced in the cage at seven days to both minimize stress from novel objects and maintain a sanitary environment.

#### Social defeat (SD)

Repeated SD in dyadic dominant/subordinate conflict housing was used to induce alterations of behavioral affect in intruder mice. Briefly, and as described previously (Lehmann and Herkenham, 2011), dominant aggressor CD-1 male mice were single-housed in a polycarbonate cage (24.0 cm×46.0 cm×15.5 cm, Lab Products Inc., Seaford, DE) for one week. Experimental intruder C57BL/6 male mice were subsequently placed into the resident CD-1 mouse’s home cage into which a 1/8-inch thick perforated transparent polycarbonate partition had been placed down the middle to separate the pair. The partition was removed for 5–10 min allowing agonistic encounters between the pair. Interaction periods were videotaped and analyzed for aggressive, submissive, and exploratory behaviors by the subordinate intruder mouse. Mice were exposed to either a 10-min single defeat session (acute) or to daily 5-min defeats for 14 consecutive days (chronic).

### Restraint Stress (RS)

HC-housed mice were placed into a 50-mL conical Falcon tube with holes drilled for adequate ventilation for three h/day for two weeks. This form of restraint has been demonstrated to induce a depressive-like phenotype [51].

### Tube Test for Dominance Hierarchy

The tube test, in which one mouse forces an opponent mouse to retreat and back out of a narrow tube, has been used to measure dominance status in mice [52], [53]. Two mice from the same cage were placed at opposite ends of a 30 cm-long×2.8 cm-ID polycarbonate tube. Once each mouse willingly advanced so that the entire body of the mouse was inside the tube, perforated dividers, each placed 12.5 cm from the ends of the tube, were removed. The mice explored in a forward direction until meeting head-to-head in the middle, where one mouse forced the other mouse to retreat and back out of the tube. The mouse that forced the other mouse out was identified as the winner, or dominant of the two, and the retreater was identified as the loser, or subordinate of the two. The test was repeated using a round-robin design such that each mouse was matched with every other mouse in the same home cage once. Mice received a score based on how many tube test matches it won that day (0–3). The average score after two weeks of testing done once every other day was used to determine the cage hierarchy.

### Social Interaction Task

Mice were placed in an open-field box (46 cm×46 cm) containing two upside-down wire cages. Both served as novel objects, and one contained the aggressor CD-1 mouse used during SD. Test mice were placed in the center of the open field and allowed to explore for 30 min. Sessions were videotaped and later analyzed using automated behavioral tracking software (TopScan/ObjectScan, CleverSystems). Social interaction quotients following the interaction task were quantified as a ratio between duration investigating the aggressor CD-1 mouse and the novel object.

### Tail Suspension Test (TST)

The TST consisted of securing the mouse by the tip of its tail using adhesive tape 60 cm above the floor for six min. The last five min were videotaped from the side and automatically scored for time spent immobile (no movement of hindlimbs) (Tail Suspension Scan, CleverSystems Inc.).

### Forced Swim Test (FST)

For the FST, mice were placed in a transparent cylindrical tank (30 cm high, 10.2 cm diameter) filled to a depth of 18–22 cm with water at 28–30°C. The last five min of a six-min session were videotaped and time spent immobile automatically calculated (Forced Swim Scan, CleverSystems Inc.).

### Sucrose Preference Test (SPT)

Mice were subjected to a two-bottle choice sucrose (1%) preference test. Testing was carried out over three days in the home cage. Each mouse was given simultaneous access to two dual ball sipper-top bottles (AnaCare, Potomac, MD, USA), one with purified water and one containing a 1% sucrose (Sigma Aldrich, St Louis, MO, USA) solution ad libitum. The amounts consumed over the whole three-day period were measured and the preference for the sucrose solution was calculated according to the formula: percentage of preference = ((sucrose solution intake/total intake) × 100%).

### Statistics

Data for all experiments were analyzed using parametric statistics with ANOVA, mixed-model repeated measures ANOVA, or multi-factor ANOVA as appropriate using SPSS software. ANOVA analysis was followed by *post hoc* tests or planned comparisons as projected from the design of each experiment. Bivariate correlations were determined using Spearman’s or Pearson’s correlation coefficients.

## Results

### Optimization of Experimental Conditions for USM in Male Mice

The first experiment examined the effects of trial length on exploratory and urine-marking behaviors of home-cage housed (HC) male mice exposed to an open-field arena that was spotted in one region with proestrus female urine. A binarized image of urine deposits made by a male after a 10-min trial is shown in Figure 1a. As shown in Figure 1b, male mice displayed a significant preference for marking the quadrant containing female urine (area within 10 cm of the female urine spot) for all three tested trial lengths (one way ANOVA; 5 min, F _3,47_ = 3.77, p<0.01; 10 min, F _3,39_ = 8.37, p<0.0005; 30 min, F _3,39_ = 5.21, p<0.005). Although ANOVA detected a significant effect for each timed trial, only during the 10-min trial were marking preferences for the female urine significantly higher vs. all other regions (*post hoc* p<0.05). The area of urine marks in the total arena increased significantly across trial length, (Fig. 1b insert; F _2,31_ = 4.23, p = 0.0001), and during the 30-min trial, males showed an increased tendency to mark the perimeter of the open field. This perimeter marking may have masked preference for marking female urine.

A time-course analysis of the duration spent in each of the four quadrants was performed for the 5 (Fig. 1c), 10 (Fig. 1d), and 30 min (Fig. 1f) trial periods. Males showed a significant preference for the female quadrant compared to all other quadrants (Interaction; 5 min, F _12,176_ = 2.18, p<0.01; 10 min, F _27,324_ = 1.67, p<0.02; 30 min, F _87,1044_ = 1.3, p<0.03) during the first min of the trial period only (*post hoc* p<0.05). This effect was observed independent of trial length. Males also interacted with the urine spot directly and showed a strong proclivity for sniffing the female urine spot during the first min of the trial exposure period (time spent sniffing urine spot during the 1^st^ min = 8.5±0.9 sec, 11.6±1.3 sec, and 11.4±3.9 sec for the 5, 10 and 30 min trial periods respectively). However this effect waned rapidly such that the first min accounted for a sizable portion of the total time spent investigating the urine spot (total time sniffing urine spot = 20.1±3.7 sec, 39±3.4 sec, and 91.4±8.1 sec for the 5, 10 and 30 min trial periods respectively). Thus, preferential exploration of female urine and scent marking appears to be an early event within the trial following initial discovery of the female urine spot.

Given these findings, we determined that 10-min trial durations provide the optimal conditions for analyzing female urine marking preference, and these parameters were utilized in the next three experiments.

### Specificity for Marking Male and Female Urine

**Figure 2:**
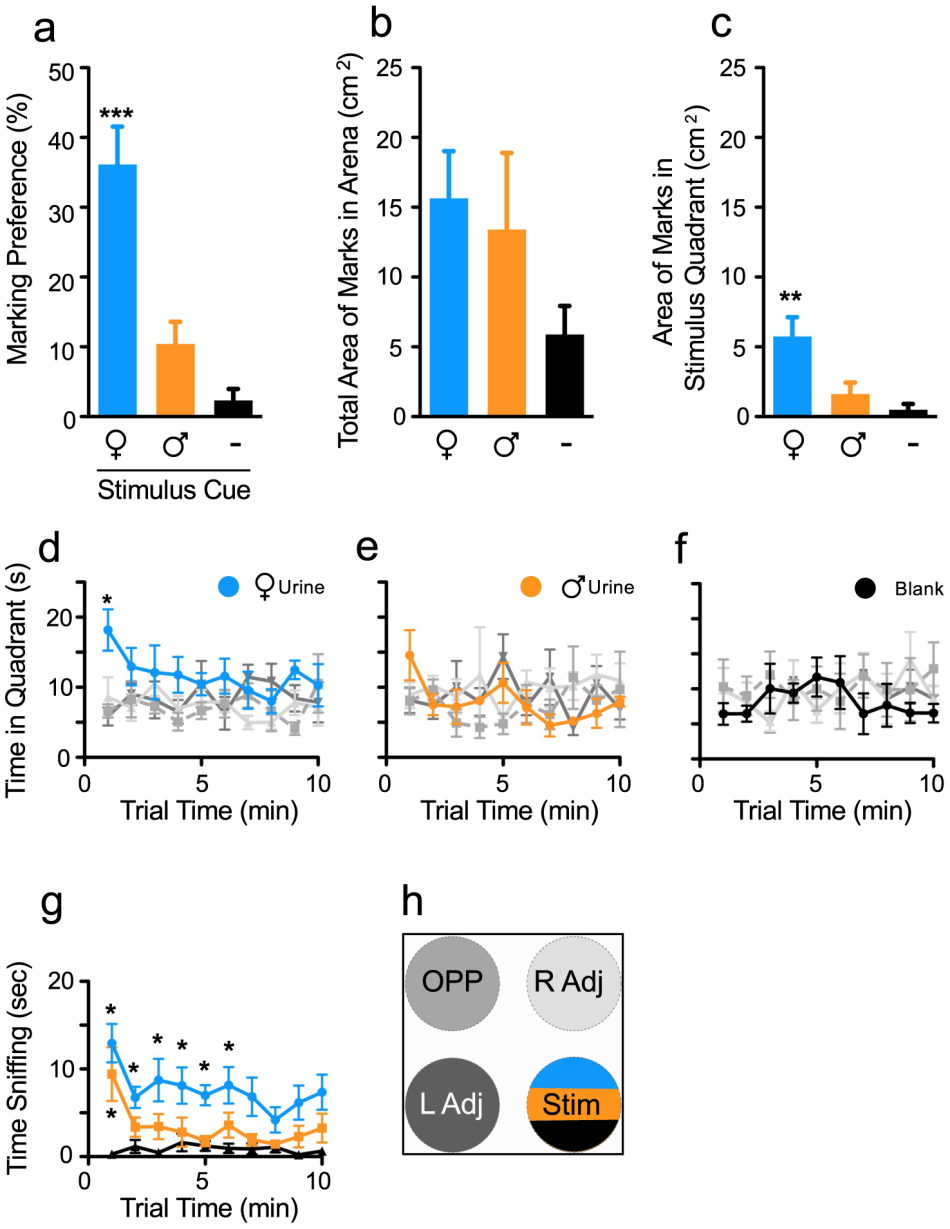
Scent marking behaviors are dependent on the sex of the urine-donor mouse. This urine scent-marking test used proestrous female urine, male urine, or blank control as the stimulus cue. **a)** Adult male mice show a significant preference for marking proestrous female but not male urine or blank control. b**)** Although the total area of urine scent marks in the arena was not affected by the stimulus cue, **c)** female urine elicited a stronger scent marking response compared to male urine or blank controls and males preferentially deposited more urine scent marks in the stimulus (Stim) quadrant when proestrous female urine was present. Time course analysis of quadrant preference are shown for tests that used **d)** proestrous female urine, **e)** male urine, or **f)** blank as a stimulus cue. **g)** Males spent more time investigating proestrous female urine compared to male urine or blank control. **h)** Legend for line graphs indicating quadrants and location. Bars represent mean+SE (n = 8 per group), *p<0.05, **p<0.01, ***p<0.001.

We next compared the preference of adult males to mark urine from conspecific males or proestrous females. Blank paper was used as a control. Males showed a substantially robust preference for marking female urine (Fig. 2a; F _2,23_ = 26.46, p<0.0001). The total area of urine marks in the arena was not significantly altered between groups (p<0.41), although arenas with male or female urine stimulus cues tended to show more marking compared to blank arenas (Fig. 2b). However, males deposited significantly more urine proximal to the female urine than male urine or to blank control spot (Fig. 2c; F _2,23_ = 7.69, p<0.005).

Similar to the first experiment, males showed a strong preference for exploring the quadrant with female urine, but this effect was evident during the first min only (Fig. 2d; quadrant preference, F _3,252_ = 7.95, p<0.01). In contrast, no significant quadrant preference was detected in tests where either male urine (Fig. 2e; quadrant preference, F _3,252_ = 1.02 p = 0.22) or no stimulus (Fig. 2f, quadrant preference, F _3,252_ = 0.39 p = 0.76) was used. Males did show increased interest towards sniffing either female or male urine spot directly compared to sniffing blank control spots (Fig. 2g; stimulus cue, F _2,189_ = 25.86, p<0.0001, time effect, F _9,189_ = 2.68 p = 0.01). This effect was significant during the first 6 min of tests using female urine and during the first min when male urine was used (p<0.05 compared to control conditions). However, the males’ interest in sniffing urine or preference for the urine quadrant was not correlated with scent marking responses (p>0.05).

### Validating the USM Test: Effects of Stress and Environmental Enrichment on USM

**Figure 3:**
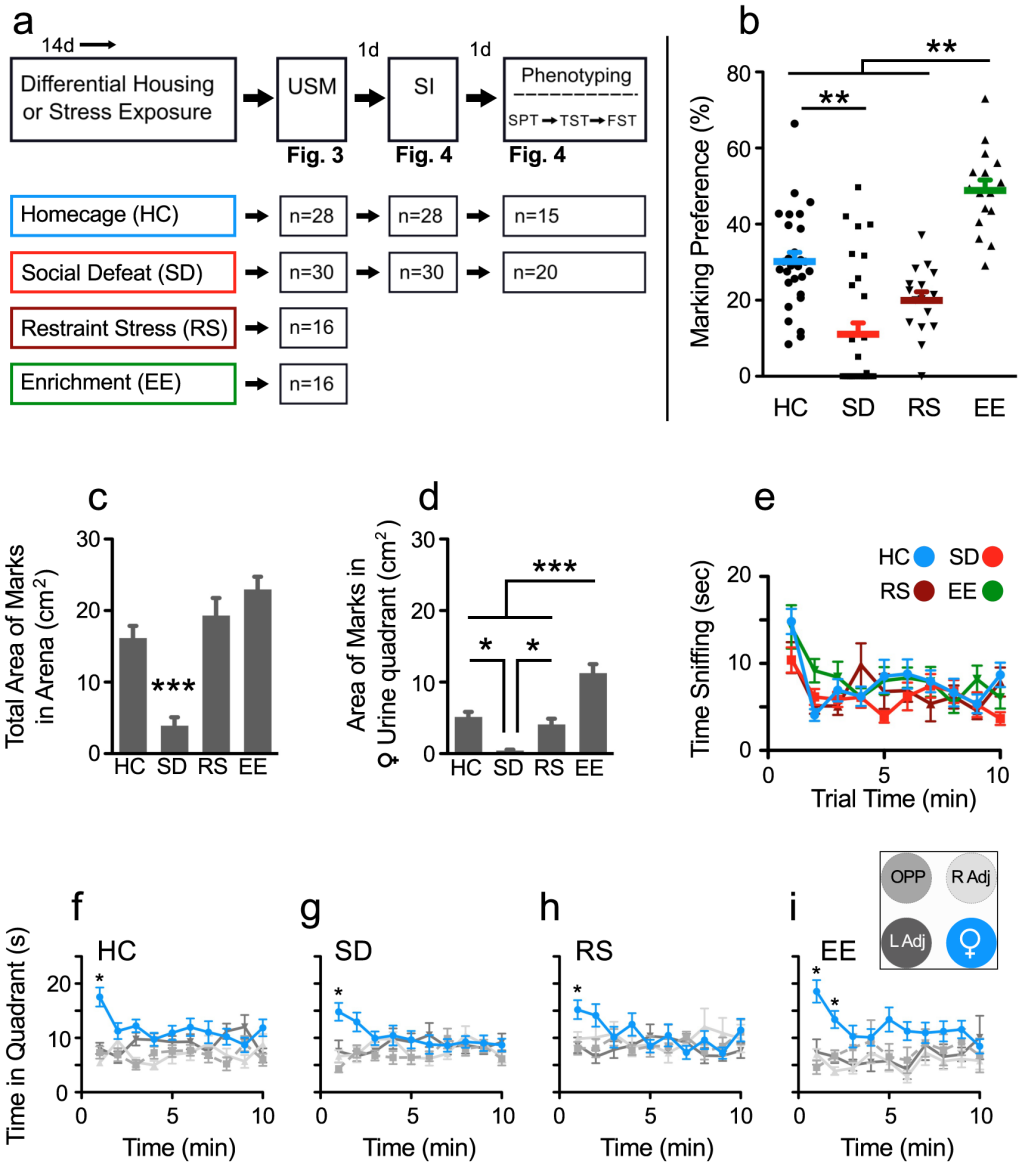
Enriched housing environments and psychological stress have divergent effects on scent marking behaviors. **a)** Experimental design testing the effects of differential housing environments on urine scent marking, and testing the correlation between urine scent marking (USM) behaviors and depressive-like behaviors. Mice were exposed to homecage (HC), social defeat (SD), restraint stress (RS), or environmental enrichment (EE) for 14 days. All mice were then tested for scent marking behaviors. Twenty-four h later, social interaction (SI) was examined in HC and SD mice. A subset of SD and HC mice was further exposed to the saccharine preference test (SPT; 3 days), tail suspension test (TST) and forced swim test (FST). Numbers of animals used in each test are shown. **b)** EE robustly increased the male’s preference to scent mark proestrous female urine whereas SD significantly reduced marking preferences. Restraint stress (RS) had no effect compared to HC groups. **c)** The total area of scent marks deposited in the arena was significantly reduced in SD mice; no effect of restraint stress or EE was observed. **d)** The area of scent marks deposited in the quadrant with female urine was significantly elevated in EE mice and significantly reduced in SD mice compared to all other groups. **e)** All groups spent a similar amount of time investigating female urine. **f–i)** Significant preference for female urine quadrant was detected during the first trial min in mice exposed to HC (**f**), SD (**g**), restraint stress (**h)**, and during the first two min in EE (**i**) housed mice. Legend for **f–i** is shown in the insert next to **Fig. 3i**. Bars represent mean+SE (n = 28 for HC, 30 for SD, 16 for RS, and 16 for EE groups), *p<0.05, **p<0.01, ***p<0.001.

We next evaluated whether USM scores are sensitive to differential housing conditions and to a restraint-stress paradigm. Enriched housing environments (EE) enhance cognitive and sensory stimulation, reduce anxiety, and confer behavioral resilience to stress. In contrast to the beneficial effects of EE, exposure to conflict housing featuring social defeat (SD) induces pronounced maladaptive behavioral phenotypes in mice. We hypothesized that the beneficial or deleterious effects of contrasting environments on mood would be accurately tracked in the USM test. We found that after two weeks of EE housing, mice showed significant increase in marking preference compared to HC, SD, or RS. (Fig. 3b; F _3,89_ = 30.52, p<0.0001, *post hoc* p<0.05 vs. all other groups). Exposure to SD for two weeks severely reduced marking preference compared to HC (*post hoc* p<0.05). However, restraint stress (RS) had no effect on marking compared to HC mice.

Defeated mice showed significantly reduced total arena urine deposition compared to all other groups. (Fig. 3c; F _3,89_ = 16.4, p<0.0001, *post hoc* p<0.05 vs. all other groups). Total arena urine deposition was comparable between EE, HC, and RS mice. EE-housed mice showed significantly more urine marks within the quadrant containing female urine compared to all other groups (Fig. 3d; F _3,89_ = 23.32, p<0.0001, *post hoc* p<0.05 vs. all other groups). In contrast, SD mice showed a significant reduction in proximal urine deposition compared to all other groups, including RS mice (*post hoc* p<0.05 vs. all other groups), indicating that alterations in marking preference are stress-specific.

Although EE increased and SD reduced marking preferences, all groups spent a similar amount of time investigating the urine spot (Fig. 3e; Housing Effect F _3,86_ = 1.32, p = 0.11). However, the time spent investigating the urine spot was elevated during the first min period (Time effect F _9,774_ = 8.75, p<0.001) (*post hoc* p<0.05 first vs. all other time periods).

Male mice in all treatment conditions showed a significant preference for exploring the quadrant with female urine (HC, Fig. 3f; Interaction effect F _27,972_ = 2.34, p<0.005, quadrant preference F _3,108_ = 18.1, p<0.0001) (SD, Fig. 3g; Interaction effect F _27,1044_ = 2.88, p<0.005, quadrant preference F _3,116_ = 3.73, p<0.001) (RS, Fig. 3h; Interaction effect F _27,540_ = 2.03, p<0.002) (EE Fig. 3i; quadrant preference F _3,60_ = 10.52, p<0.0001). Significant place preference was detected during the first trial min in homecage, defeated, and restraint stress mice, and during the first two min in EE-housed mice (*post hoc* p<0.05 female quadrant vs. all other quadrants).

We further examined if USM preference was correlated with quadrant place preference and time sniffing female urine. Because mice showed elevated place preference and a proclivity towards investigating female urine during early trial periods, correlations were made using both the total trial time and the first trial min. However, in all of the tested housing conditions (HC, SD, RS, or EE), no correlations between urine sniffing or quadrant preference and USM preference were found (p>0.05 for all). Thus, USM behavior is dissociated from investigatory behavior, suggesting that scent marking is a display of motivated behavior of core importance to the animal.

### The USM Test and Resilience or Susceptibility

**Figure 4:**
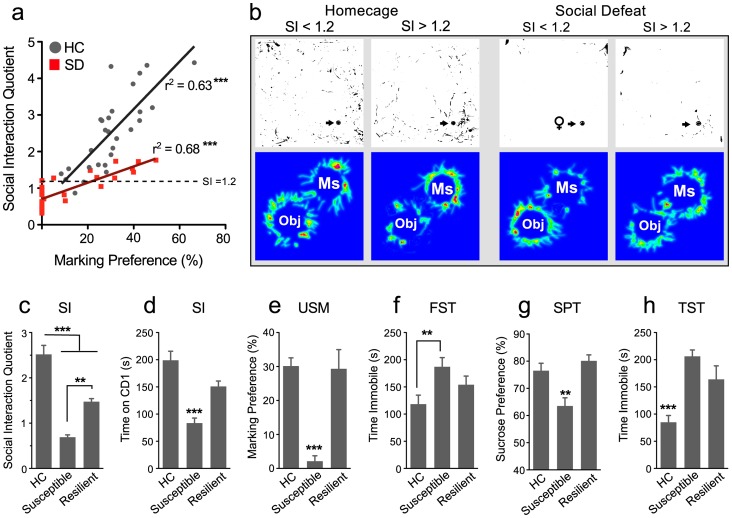
Scent marking behaviors are highly correlated with social interaction of an aggressor mouse in both defeated and non-defeated male mice. The social interaction (SI) quotient is determined by proportion of time the experimental mouse explored an enclosure containing an aggressor mouse vs. an empty enclosure. a) Urine scent marking preferences positively correlated with SI scores in both home cage (HC) and socially defeated (SD) mice (Pearson’s r^2^, *P*<0.001). Defeated mice with SI scores ≥ 1.2, denoted by the dotted line, were deemed resilient to defeat stress. (n = 30 for SD and 28 for HC) b) Examples of data plots illustrate that SI scores and marking preferences within treatment groups were correlated and further demonstrate differences in both behaviors between HC and SD mice. c) In response to a CD-1 social target mouse, both Resilient and Susceptible mice showed reduced SI scores compared to HC mice. However, SI scores were significantly higher in Resilient than Susceptible mice. d) Only Susceptible mice avoided interacting with the CD-1 mouse and e) displayed reduced USM preference. f) Similarly, only Susceptible mice showed increased immobility in the forced swim test (FST) and g) displayed anhedonia as measured by a reduction I 1% sucrose preference test (SPT). h) Both defeated groups displayed increased immobility in the tail-suspension test (TST). Bars represent mean+SE (n = 15 for HC, 7 for resilient, and 13 for susceptible groups) **p<0.01, ***p<0.001.

Studies have shown that mice exposed to chronic SD display a reduction in social interaction (SI), which is measured by comparing the time a mouse spends interacting with a social target to the time spent investigating an empty target enclosure. We used this test as a benchmark test to validate the USM measures (Fig. 4a) and found a significant positive correlation between SI and marking preference in SD mice (r^2^ = 0.68, p<0.0001, n = 30). A similar correlation was also detected in HC mice (r^2^ = 0.63, p<0.0005, n = 28). The slope of the linear regression line is significantly higher in the HC group (F _1,54_ = 11.28, p<0.001), signifying that SI scores increase more rapidly with USM preferences in the HC groups than in the SD group. Despite an overall reduction in social interaction, socially defeated mice showed a large variation in SI scores. Because most HC mice prefer interacting with a social target than an empty enclosure, an SI score of 1.2 was set as a limit to differentiate between mice that we defined as susceptible to SD (SI < 1.2) and those that were resilient (SI ≥ 1.2) (Fig. 4a). Examples of data plots (Fig. 4b) illustrate how HC and SD mice that spend more time interacting with a social target also show a strong preference for marking female urine. Although both subgroups of defeated mice showed reduced SI scores compared to HC mice (Fig. 4c; F _2,57_ = 35.46, p<0.0001, *post hoc* p<0.01), SI scores for resilient mice were significantly higher than susceptible mice (*post* hoc, p<0.001). However, only susceptible mice spent significantly less time interacting with a social target (Fig. 4d; F _2,57_ = 17.79, p<0.0001, *post hoc* p<0.01). Similarly, both HC and resilient mice display similar and elevated USM preferences compared to susceptible mice (Fig. 4e; F _2,57_ = 31.37, p<0.0001, *post hoc* p<0.001).

We examined whether resilience to defeat-induced changes in USM and SI generalized to other behavioral measures of behavioral despair and anhedonia. Only susceptible mice displayed a significant decrease in FST mobility (Fig. 4f; F _2,34_ = 4.76, p<0.05, *post hoc* p<0.05) and sucrose preference (Fig. 4g; F _2,34_ = 7.65, p<0.005, *post hoc* p<0.01), consistent with increased depression-like behavior. However both subgroups demonstrated decreased mobility in the TST (Fig. 4h; F _2,34_ = 21.24, p<0.001, *post hoc* p<0.01).

We further evaluated whether USM preferences correlated with the other behavioral measures (Table 1). Collectively, the data demonstrated that USM preferences in both HC and SD animals positively correlated with other measures of hedonic behavior, i.e., social interaction and sucrose preference, but not with immobility in the forced swim and tail suspension tests. However, displays of hedonic behaviors did not correlate with the time sniffing urine and time spent in the female urine quadrant in either defeated or HC mice (p>0.05).

**Table 1:** Urine scent marking preference positively correlate with hedonic behaviors.

	Exp 3: Stress Effects		Exp 4: Pharmacological rescue			
	Marking Preference		Marking Preference			
	HC	SD	HC+Sal	HC+Flx	SD+Sal	SD+Flx
Social Interaction	0.867 (0.0001)	0.841 (0.0001)	0.571 (0.18)	0.783 (0.037)	0.824 (0.012)	0.71 (0.041)
FST Immobility	0.336 (0.221)	−0.098 (0.672)	0.004 (0.994)	−0.318 (0.486)	−0.638 (0.089)	−0.655 (0.078)
TST Immobility	−0.137 (0.669)	0.053 (0.819)	0.136 (0.772)	0.332 (0.467)	−0.689 (0.059)	−0.519 (0.187)
SPT	0.541 (0.03)	0.743 (0.0002)	0.594 (0.16)	0.764 (0.046)	0.534 (0.113)	0.702 (0.042)

This table shows the correlation between marking preferences and four other tests used to assess depressive-like behavior (FST, forced swim test; TST, tail suspension test; SPT, sucrose preference test) from data collected in Experiment 3, Stress Effects, and Experiment 4, Pharmacological Rescue. (HC, home cage; SD, social defeat; Sal, saline vehicle; Flx, fluoxetine). Data is presented as Pearson’s r (Significance, 2-tailed).

### Pharmacological Rescue of Behavioral Pathology in Socially Defeated Mice

**Figure 5:**
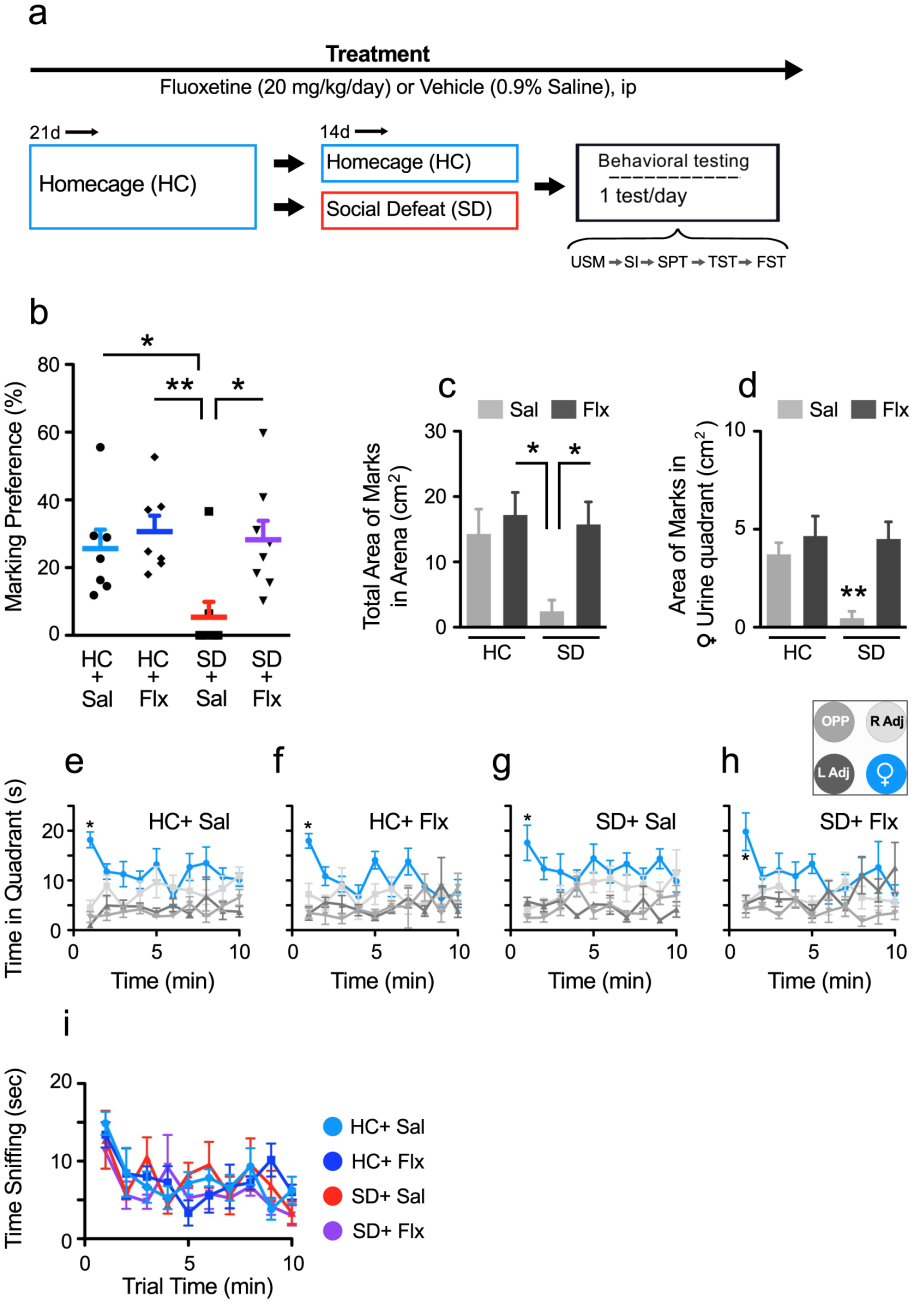
Fluoxetine prevents a decline in urine scent marking (USM) preferences induced by social defeat. **a)** Diagram illustrates experimental groups and study design. Fluoxetine (Flx) or saline vehicle (Sal) was administered once a day for the duration of the experiment. **b)** Saline treated defeated mice show substantial reductions in USM preferences compared to all other groups. Mice treated with fluoxetine show USM preferences similar to non-defeated groups. **c–d)** Fluoxetine completely reverses the decline in the area of urine marks in the arena **(c)** and in the female urine quadrant **(d)** induced by social defeat. **e–h)** Time spent in each quadrant for each treatment condition is shown. All groups show significant preference for the female urine quadrant during the first trial min. **i)** Interest for sniffing female urine was elevated early in the trial period (Time effect; F _9,216_ = 4.89, p<0.0001), no effect of treatment was observed. (HC; homecage, SD; social defeat) Bars represent mean+SE (n = 7 for HC groups, 8 for SD groups) *P<0.05, **P<0.01.

Chronic treatment with antidepressants can remediate maladaptive behaviors in socially defeated mice, and we tested whether USM preferences were similarly malleable (Fig. 5a). We observed that fluoxetine administered daily prior to and during SD protected against the emergence of depressive behaviors. Chronic fluoxetine completely reversed the decline in USM preference induced by SD (Fig. 5b; stress effect F _1,26_ = 4.81, p<0.03 and drug effect F _1,26_ = 7.31, p<0.01). USM preference was significantly attenuated in vehicle-treated defeated mice compared to all other groups (*post hoc* p<0.01). The strong differences in USM preferences in saline- vs. fluoxetine-treated defeated mice were seen in marking behavior: the area of urine marks in both the arena (Fig. 5c; stress effect F _1,26_ = 4.43, p<0.05 and drug effect F _1,26_ = 6.55, p<0.02) and within the female-urine quadrant (Fig. 5d; interaction effect F _1,26_ = 4.37, p<0.04, stress effect F _1,26_ = 5.25, p<0.03, and drug effect F _1,26_ = 4.37, p<0.002) were substantially elevated in fluoxetine-treated compared to vehicle-treated defeated mice (*post hoc* p<0.01). In contrast, all treatment groups showed preference for the female urine quadrant during the first min of exposure (Fig. 5 e–h) (HC+Sal, Fig. 5e; Interaction effect F _27,216_ = 1.67, p<0.02, quadrant preference F _3,27_ = 21.7, p<0.0001) (HC+Flx, Fig. 5f; Interaction effect F _27,216_ = 1.69, p<0.03, quadrant preference F _3,27_ = 14.4, p<0.0001) (SD+Sal, Fig. 5g; quadrant preference F _3,27_ = 24.1, p<0.0001) (SD+Flx, Fig. 5h; quadrant preference F _3,27_ = 8.05, p<0.001)(All treatment groups; *post hoc* test, p<0.01 female urine quadrant vs. all other quadrants during 1^st^ min.). In addition, all groups spent a similar amount of time sniffing female urine directly (Fig. 5i; p>0.05). Quadrant preferences and interest in female urine remained independent of USM behaviors (p>0.05 for all groups).

**Figure 6:**
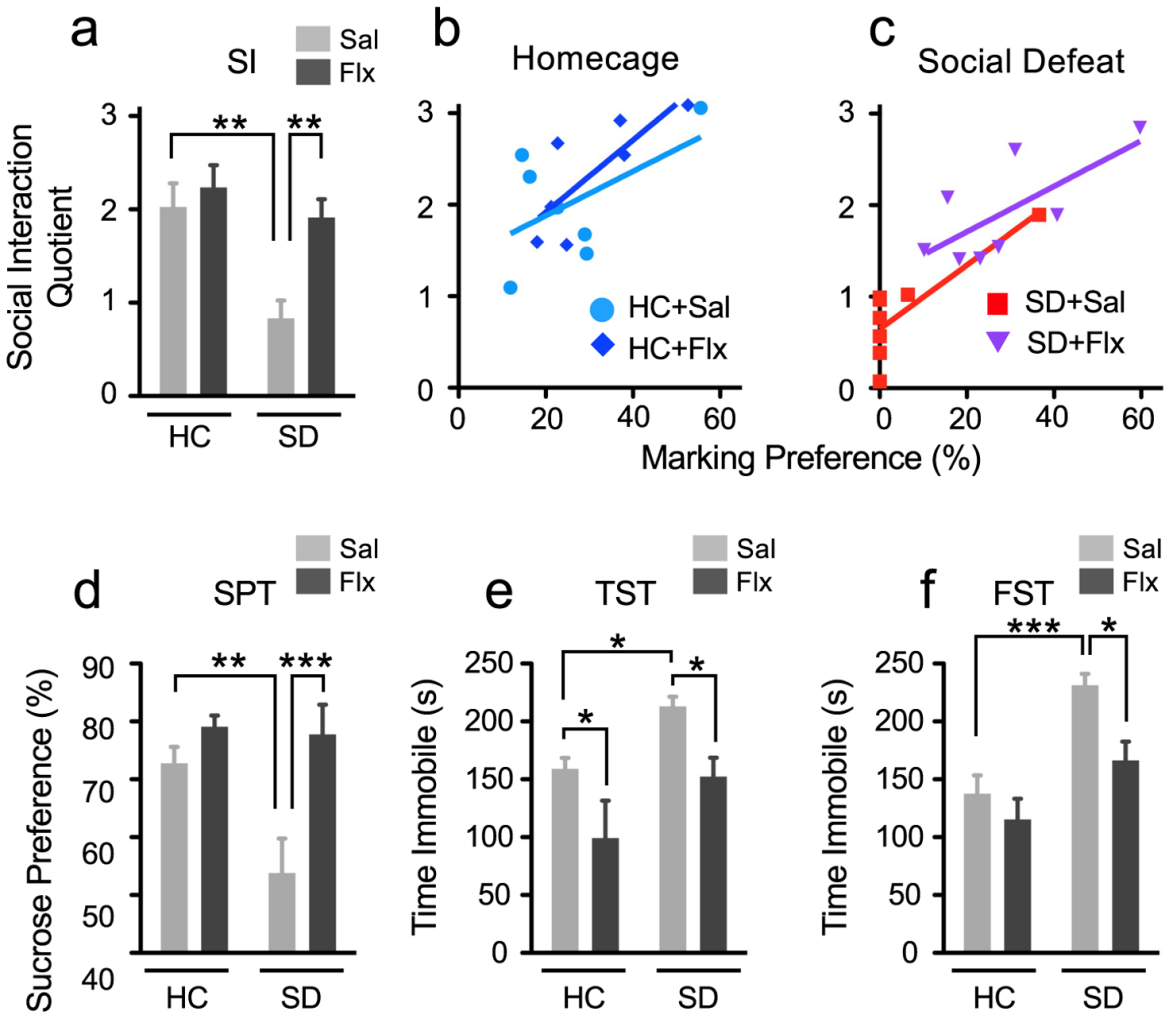
Chronic treatment with fluoxetine protects against the emergence of depressive behaviors induced by social defeat. **a)** Fluoxetine reverses social avoidance induced by chronic social defeat. **b)** Linear regression analysis revealing a significant correlation between social interaction and urine scent marking (USM) preferences in homecage mice treated with fluoxetine, but not in homecage mice treated with saline vehicle. **c)** Social interaction scores and USM preferences strongly correlate in defeated mice treated with either saline vehicle or fluoxetine. **d)** Fluoxetine prevents anhedonia, measured by a reduction in sucrose preference (SPT), induced by social defeat. **e)** Fluoxetine reduces the time spent immobile during the tail suspension test (TST). **f)** In the forced swim test (FST), elevations in floating time induced by social defeat are blocked by fluoxetine. (HC; homecage, SD; social defeat, Sal; saline vehicle, Flx; fluoxetine) Bars represent mean+SE (n = 7 for HC groups, 8 for SD groups) *P<0.05, **P<0.01, ***P<0.001.

Chronic treatment with fluoxetine also reversed social avoidance induced by social defeat (Fig. 6a; stress effect F _1,26_ = 12.02, p<0.001, and drug effect F _1,26_ = 8.69, p<0.005). In addition to reversing social avoidance, fluoxetine also protected against a decline in sucrose preference induced by social defeat (Fig. 6d; stress effect F _1,26_ = 5.1, p<0.03, drug effect F _1,26_ = 9.8, p<0.005) (*post hoc* p<0.001).

Defeated mice treated with fluoxetine further showed decreased immobility in the tail suspension test (Fig. 6e; stress effect F _1,26_ = 9.9, p<0.004, drug effect F _1,26_ = 12.7, p<0.001) (*post hoc* p<0.01) and forced swim test (Fig. 6f; stress effect F _1,26_ = 22.2, p<0.001, drug effect F _1,26_ = 8.05, p<0.01), demonstrating the antidepressant effect of drug treatment.

Individual preferences for interacting with a social target were highly correlated with USM preferences in both fluoxetine- and saline-treated defeated mice (Fig. 6c). Similar correlations were detected in homecage fluoxetine-treated mice but not in vehicle-treated mice (Fig. 6b) (Pearson’s r shown in Table 1). SM preferences strongly correlated with social interaction and sucrose preference, but not with immobility in the FST and TST, in fluoxetine-treated animals Table 1). Correlations between USM and social interaction were also detected in vehicle-treated homecage and defeated mice (summarized in Table 1), providing further validation that USM behavior reflect mood state.

### Urine Marking as a Predictor of Stress Resiliency

**Figure 7:**
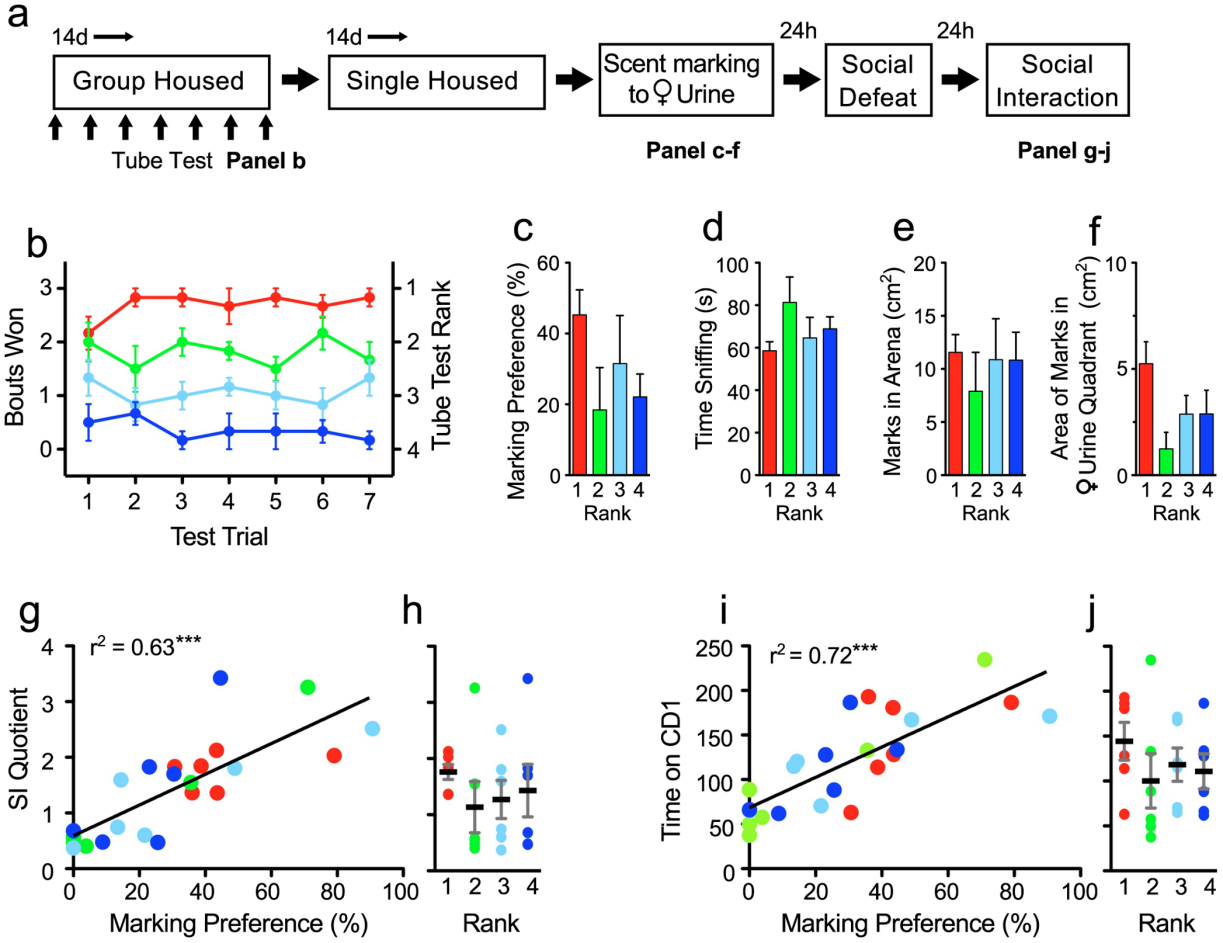
Urine scent marking preference predicts resiliency to acute social defeat. **a)** Diagram illustrates the study design. Social rank in group-housed mice was determined using the tube test over two weeks **(b)**. Mice were then single housed, and scent marking behaviors were examined **(c–f)**. Subsequently mice were exposed to social defeat (SD), and social interaction (SI) behaviors were examined 24 h later **(g–j)**. **b)** Social hierarchy remained stable during the duration of testing. The average rank position (right axis) for 6 cages was assigned post test completion by averaging the number of tube test bouts won (left axis) across all testing days. Ranks 1 to 4 correspond with most dominant to most subordinate. Low dominance rank had no effect on marking behaviors including: **c)** USM preference, **d)** time spent sniffing female urine, **e)** total area of scent marks in arena, or **f)** area of scent marks within 10 cm of proestrous female urine spot. USM preferences observed prior to acute SD exposure positively correlated with SI scores observed after SD (Pearson’s r^2^, *P*<0.001). **h)** Social ranking had no effect on SI scores. **i)** USM preferences positively correlated with social interaction time (Pearson’s r^2^, *P*<0.001), a non-transformed measure of social interaction preference after SD. **j)** Social ranking had no effect on time interacting with the CD-1 social target mouse. Bars represent mean+SE (n = 24, 6 mice per rank), ***p<0.001 (n = 24).

We were struck by the fact that USM and social interaction scores correlated even in unstressed HC mice. We questioned whether mice that were more elevated in the hierarchy that occurs in group housing conditions would show relatively higher marking preference in the USM test. Because male mice were group-housed four-per-cage prior to the start of experimental testing, and because co-housed male mice are known to form dominance hierarchies [52], we hypothesized that social interactions during the establishment of these hierarchies may contribute to variations in the social interaction and scent marking. We were further interested to see whether USM scores were predictive of later responses to a single exposure to SD stress, and whether social standing in a hierarchy correlated with the USM scores and the stress response measured in the social interaction task. First, mice were group-housed for 14 days, and the hierarchy was measured in the tube test. Next, mice were singly housed for two weeks and then USM tested, exposed to a single SD session, and tested for resilience in the SI task (experimental design is shown in Fig. 7a).

The tube test was used to determine hierarchical status in six group cages. We observed a strikingly stable hierarchical structure within the groups (Fig. 7b). A transitive and linear social hierarchy (A>B>C>D) was observed during 88.1% of the tube-test trials (37 out of 42 trials). Dominance within a cage was highly stable across trials, and in 85.7% of trials, the dominant mouse remained dominant during subsequent trials (36/42). A greater variation in ranking was observed for less-dominant mice; only in 52.4% of trials was the order of hierarchy in a given four-mouse group conserved during subsequent trials (22/42). Ranking was assigned post-test by averaging number of tube-test bouts won across all testing days. The most dominant mouse was assigned a rank of 1.

Next, mice were singly housed for two weeks prior to USM testing in order to reduce the effects of agonistic interactions on subsequent behaviors, and to mirror HC conditions of the previous three experiments. Mice showed USM preferences comparable to previously tested HC groups (29.32±5.24%). However, hierarchical ranking did not correlate with any of the examined USM measures including marking preference (Fig. 7c; r = −0.25, p = 0.23 ), time spent investigating female urine during the total trial period (Fig. 7d; r = 0.07, p = 0.71), or during the 1^st^ trial min (data not shown; r = −0.09, p = 0.67). Furthermore, the total area of marks in the arena (Fig. 7e; r = 0.01, p = 0.95), or area of marks within 10 cm of proestrous female urine (Fig. 7f; r = −0.23, p = 0.26) did not correlate with rank hierarchy. Mice showed a significant quadrant preference during the first trial min only (F _3,95_ = 2.89, p<0.05), and spent significantly more time in the female urine quadrant (15.69±3.57 sec) compared to all other quadrants (8.16±1.65, 6.69±1.26, 9.44±2.11 sec for Opp., Adj L., and Adj R. quadrants respectively) (*post hoc* female vs. all other quadrants, p<0.05 ). However this effect was independent of hierarchical rank (r = −0.193, p = 0.52).

Resilience to social defeat was assessed during the social interaction task following the SD session. Both the social interaction quotient (Fig. 7g and 7h) and time the defeated mouse spent interacting with the aggressor-containing enclosure (Fig. 7i and 7j) were scored. No correlation was detected between cage rank and either the social interaction quotient (Fig. 7h; r = −0.11, p = 0.61) or time spent interacting with the CD-1 mouse (Fig. 7j; r = −0.16, p = 0.44). However USM preferences scored prior to defeat positively correlated with social interaction preference after defeat (Fig. 7g; r = 0.79, p<0.0001). Similarly, mice with high USM preferences spent significantly more time interacting with the aggressor CD-1 mouse after defeat exposure (Fig. 7i; r = 0.85, p<0.0001, suggesting that scent-marking behaviors can predict stress resiliency to social defeat.

## Discussion

Urine scent marking (USM) has an ethologically important role in social communication in rodents, and it is highly adaptive and sensitive to environmental cues and social status [54], [55]. We hypothesized that male USM behavior offers a naturalistic measure of social motivation that can be used to evaluate hedonic behaviors relevant to the study of mood disorders. We here demonstrated that, in male mice, preference for marking proestrous female urine provides a novel phenotype for assessing depressive-like behaviors and can further be used to predict changes in behavior induced by social defeat (SD) stress. We showed that adult male mice display a strong preference for marking proestrous female urine with a high degree of specificity (Figs. 1, 2). We then showed that exposure to chronic SD but not chronic restraint stress robustly decreased USM preferences whereas exposure to environmental enrichment (EE) increased them (Fig. 3). We showed further that chronic fluoxetine reversed declines in USM and other behavioral abnormalities induced by social defeat, a response consistent with the antidepressant-like effects of this drug (Figs. 5, 6). We established that USM behavior closely correlated with other hedonic measures but not with measures of behavioral despair (Table 1). Finally, we found that USM did not correlate with the hierarchical status of mice in group-housed homecage conditions (Fig. 7).

We also showed that USM scores correlated with susceptibility to the maladaptive consequences of SD stress (Fig. 4). Many individuals exposed to chronic stress develop affective disorders, but some remain resilient [8]. The large variance in behavioral outcomes after SD was also observed in USM behaviors–mice resilient to SD, i.e., those that showed high social interaction scores, showed relatively high scent marking compared to mice with low social interaction scores. Although the majority of mice exposed to SD showed a sharp reduction in scent marking, some showed marking behavior comparable to non-defeated mice. Numerous reports using the social interaction test have observed a similar spectrum of behavioral outcomes in defeated mice [8], [26], [56]. Social interaction is a reward-related behavior [26] and is measured by comparing the time a mouse spends in an interaction zone with a social target to the time in that zone in the absence of a social target. Susceptible mice actively avoid the social target whereas resilient mice actively investigate the social target. Resilience to defeat-induced social avoidance generalizes to other behavioral measures, and resilient mice show immunity to several depression-like changes (e.g., anhedonia) [8], [9], [15], [57].

We further showed that USM scores in non-stressed mice predicted behavioral outcomes after SD exposure (Fig. 7g, i), and thus mice that displayed high preference for marking female urine prior to SD showed behavioral resiliency after SD. Therefore, the USM test has predictive as well as face validity.

Our observations that adult male mice showed a strong preference for marking pro-estrous female urine but showed little response to urine from conspecific males are consistent with previous reports [27], [30]–[32], [58]. Males also spent more time investigating urine from proestrous females than from males. We utilized proestrous female urine for USM because males preferentially mark urine from proestrous than from diestrous females [33], [59]. Adult male mice smell female urine to recognize the reproductive state of potential mates [30], and they scent mark in part to promote sexual interactions. Thus, male scent marking of female urine is a sexually motivated behavioral response that serves as a sexual advertisement directed towards receptive females.

Male mice show a strong interest for investigating estrous female urine as they do for investigating other rewarding stimuli [60]. Investigation of estrous female urine activates brain regions associated with reward circuitry and elevates dopamine levels in the nucleus accumbens [34], [61], responses that are comparable to those observed during the consummation of other rewarding stimuli such as food, sugar, or psychostimulants [62]. Therefore, male USM is an appetitive behavioral response to a rewarding stimulus–estrous female urine–and brings a male animal into contact with a receptive mate, leading to the consummatory act of copulation.

These displays of sexual behavior are androgen-dependent [63], [64]. EE and SD have opposing effects on androgen levels [65] [66]. Androgens may control the divergent effects of SD and EE on USM preferences. However, further experiments will be necessary to determine a causal relationship.

Territorial urine marking is altered by subordinate status [28], [67], and repeated social defeat has been shown to reduce territorial scent marking when defeated mice are pair-housed with non-defeated males [35]. In these experiments, territorial urine marking behavior was examined either in homecage environments, in the absence of any other cues, or marking behavior was examined when defeated mice were exposed to non-defeated male mice through a perforated divider. The current experiments are the first demonstration that social defeat reduces scent marking to proestrous female urine. USM to female urine is perhaps a more relevant measure of anhedonia because male rodents perceive female urine as a rewarding cue [34]. Surprisingly, the time spent investigating female urine was not altered by any of the stress treatments nor affected by the animals’ home-cage environment (EE vs. HC) or by fluoxetine treatment. This lack of effect stands in contrast to a recent report by Malkesman et al. [34] showing that mice exposed to a learned helplessness paradigm spent less time investigating estrus female urine. The discrepancy between this report and our finding may be due to the different stress paradigms (learned helplessness vs. SD) and experimental design parameters. The female urine sniffing (FUS) test [34] tested mice in their homecages where they were habituated to the environment. In contrast, we placed into a large open field mice naïve to the experimental apparatus. The higher levels of behavioral arousal in the novel open field may affect sniffing behaviors. Further experiments are necessary to test this possibility. However, the validity of USM test for elucidating hedonic drive is supported by the positive correlation between USM preferences and other measures of hedonic behaviors in both HC and SD mice. Our data suggest that scent marking, not time spent investigating female urine, is the salient measurement for change in behavioral affect after social stress exposure. In terms of behavioral significance, scent marking leaves a lasting imprint, advertises the males’ presence to other mice, and more likely will allow fulfillment of an appetitive drive, in this case reproduction. Scent marking is clearly more consequential than sniffing choices. The observation that defeated males spend a similar amount of time investigating female urine as do non-defeated mice, but do not mark it, underscores the differences between the two behaviors.

We showed that USM is a sensitive indicator of the deleterious effects of SD but not restraint stress on mood-related behaviors. Social defeat is an ethologically relevant stress that is thought to play an important role in the onset of a variety of human psychopathologies including depression, anxiety, and post-traumatic stress disorder [12], [68]–[71]. We and others have shown that as mice accumulate social defeats, profound and stable behavioral changes reminiscent of depression occur [8], [12], [57], [71]. The differential effects of social defeat and restraint stress on USM behavior may be explained by the fact that different stressors engage distinct circuits in the brain [72]. For instance, the ability of psychogenic stress to affect scent marking behavior may be driven by the expression of corticotrophin releasing hormone (CRH) in Barrington’s nucleus; a region located within the dorsal pons that coordinates the micturition reflex [73]. Studies have reported that subordinate mice show decreased territorial marking that is coincident with urine retention and increased bladder volumes [28], [35]. Social stress but not restraint stress increases levels of both CRH mRNA and protein within Barrington’s nucleus [74], and CRH has inhibitory effects on Barrington’s nucleus neurons that project to bladder motor neurons [73], [75]. These observations suggest that visceral (bladder) dysfunction and urine retention might be specific to social stress. Urine retention may also be a visceral component of a defensive response mediated by the periaqueductal gray, which has a major input to Barrington’s nucleus [76]. This region is involved with the expression of passive coping behaviors [77] typically observed in defeated mice.

It is difficult to explain the bases for the wide range of USM and SI scores that occurs in both defeated and homecage animals. Differential social phenotypes cannot be accounted for by genetic factors, and environmental factors are nearly identical within each condition, yet they emerge nevertheless in group housing [78]. It is likely that early-life experiences prior to our manipulations influenced adult behavioral responses to female urine. Indeed, experiences accrued during adolescence can have profound consequences on adult behavioral and psychological function [79], and both human and rodent studies demonstrate that stressful experiences during adolescence are potent etiological factors that contribute to the onset of adult mood disorders [80], [81]. Animals living in social environments form dominance hierarchies, which serve to minimize violence and energy expenditure, but which also have asymmetric affects on health, reproductive success, and behavior. We tested for the presence of social hierarchy in co-housed male mice using the tube test and predicted that dominant mice would show increased scent marking and exhibit behavioral resiliency to acute SD. However, dominance rank was not significantly correlated with scent marking or with resiliency. These findings contrast with earlier work from Drickamer [67] showing a strong correlation between dominance rank and urine marking behavior. In that study, dominance rank was determined via the accumulation of social victories and defeats through forced agonist encounters between singly housed males, and territorial urine marking was assessed in the absence of female urine. In our hands, the USM test was predictive of resilience to social defeat. The USM test may be insensitive to normal social hierarchy formation but sensitive to the psychological effects of social adversity.

Research in the field of depression requires ethologically relevant animal models that employ sensitive, precise, and quantifiable tests. Currently used tests of depressive-like behavior–the sucrose preference test, forced swim test, and tail suspension test–are meant to quantify anhedonic states and behavioral despair. However, the novelty and unnatural character of these tasks may be confounding factors. Furthermore, the validity of these tests to model depression remains questionable [21], [82]. However, decreased interest in pleasurable stimuli such as sex and social interactions are one of the key symptomatic criteria for major depression [83]. Thus, direct measurements of reward-related behavior such as social interaction and sexual behavior may be more appropriate assays for depressive states. We propose that male scent marking of proestrous female urine in the USM test offers a naturalistic measure of social motivation that can be used to evaluate behaviors and neural circuits relevant to the study of mood disorders.
